# A Graph Convolutional Network–Based Method for Chemical-Protein Interaction Extraction: Algorithm Development

**DOI:** 10.2196/17643

**Published:** 2020-05-19

**Authors:** Erniu Wang, Fan Wang, Zhihao Yang, Lei Wang, Yin Zhang, Hongfei Lin, Jian Wang

**Affiliations:** 1 College of Computer Science and Technology Dalian University of Technology Dalian China; 2 Beijing Institute of Health Administration and Medical Information Beijing China

**Keywords:** chemical-protein interaction, graph convolutional network, long-range syntactic, dependency structure

## Abstract

**Background:**

Extracting the interactions between chemicals and proteins from the biomedical literature is important for many biomedical tasks such as drug discovery, medicine precision, and knowledge graph construction. Several computational methods have been proposed for automatic chemical-protein interaction (CPI) extraction. However, the majority of these proposed models cannot effectively learn semantic and syntactic information from complex sentences in biomedical texts.

**Objective:**

To relieve this problem, we propose a method to effectively encode syntactic information from long text for CPI extraction.

**Methods:**

Since syntactic information can be captured from dependency graphs, graph convolutional networks (GCNs) have recently drawn increasing attention in natural language processing. To investigate the performance of a GCN on CPI extraction, this paper proposes a novel GCN-based model. The model can effectively capture sequential information and long-range syntactic relations between words by using the dependency structure of input sentences.

**Results:**

We evaluated our model on the ChemProt corpus released by BioCreative VI; it achieved an F-score of 65.17%, which is 1.07% higher than that of the state-of-the-art system proposed by Peng et al. As indicated by the significance test (*P*<.001), the improvement is significant. It indicates that our model is effective in extracting CPIs. The GCN-based model can better capture the semantic and syntactic information of the sentence compared to other models, therefore alleviating the problems associated with the complexity of biomedical literature.

**Conclusions:**

Our model can obtain more information from the dependency graph than previously proposed models. Experimental results suggest that it is competitive to state-of-the-art methods and significantly outperforms other methods on the ChemProt corpus, which is the benchmark data set for CPI extraction.

## Introduction

Biomedical literature has grown significantly with the development of biomedical technology, which contains a large amount of valuable chemical-protein interactions (CPIs). CPI extraction plays an important role in various biomedical tasks such as drug discovery, medicine precision, and knowledge graph construction [1]. With the rapidly increasing volume of biomedical literature, it becomes time-and-resource–consuming to extract CPIs from biomedical literature manually. There are some computational methods that have been successfully proposed for automatic biomedical relation extraction [2-6]. However, most previous studies focused on the extraction of drug-drug interactions, protein-protein interactions, and chemical-disease interactions; only a few attempts were developed to extract CPIs [7].

The BioCreative VI ChemProt shared task [8] created the ChemProt data set, which is used in the development of CPI extraction methods. The current CPI extraction systems can be generally divided into two categories: the traditional machine learning–based methods and the neural network–based methods. The traditional machine learning–based methods conventionally train a CPI extractor by handcrafted features [7]. The neural network–based methods can automatically learn powerful features to train a classifier, and therefore, have become a promising method for CPI extraction.

Mehryary et al [9] combined a support vector machine (SVM) and long short-term memory (LSTM) to extract CPIs and achieved a high F-score by a rich set of features. Warikoo et al [10] also exploited a set of linguistic features to train a tree kernel classifier to obtain CPIs from biomedical literature. Generally, these methods depend heavily on feature engineering. Recently, attention mechanisms have been successfully used in many natural language processing tasks, and some works have employed it in CPI extraction. Liu et al [11] aggregated an attention mechanism and gated recurrent units (GRU) to extend the LSTM model. Verga et al [12] encoded pair-wise predictions over entire abstracts by synthesizing self-attention and convolutions. Corbett and Boyle [13] employed multiple LSTM layers with unlabeled data to extract relations amongst the ChemProt corpus and achieved good performance. Peng et al [14] applied an ensemble system to extract CPIs, which consists of three individual models, including SVM, convolutional neural network (CNN), and bi-directional long short-term memory (Bi-LSTM) modules. The system achieved an F-score of 64.1% and won the top rank in the BioCreative VI ChemProt shared task.

However, most of the proposed methods only utilize the sequential information of sentences; syntactic information has not been carefully studied yet. Due to the presence of complex sentences in biomedical literature, it is difficult to effectively learn the semantic and syntactic information for some neural network–based models (eg, CNN [15], LSTM [13,16], and GRU [17]). To address this problem, we apply a graph convolutional network (GCN) [18,19] for CPI extraction. The GCN can exploit dependency structure and capture long-range syntactic relations of input sentences. Therefore, it is more effective and precise than other modules for CPI extraction.

Additionally, sentences in the biomedical literature are generally lengthy, so there is a considerable amount of irrelevant words. For example, in the sentence “Dasatinib (BMS-354825) is a novel orally bioavailable SRC/ABL inhibitor that has activity against multiple imatinib-resistant BCR-ABL isoforms in vitro that is presently showing considerable promise in early-phase clinical trials of chronic myeloid leukemia (CML),” “Dasatinib (BMS-354825) is a novel orally bioavailable SRC/ABL inhibitor” can already express the inhibitory relationship between the entities “Dasatinib” and “SRC.” Other words, which may affect the performance of the relation extractor, are irrelevant. Inspired by Zhang et al [20], we apply a path-centric pruning strategy to incorporate relevant information while maximally reducing the influence of noisy words in long sentences. This strategy retains tokens that are up to distance *N* away from the dependency path in the lowest common ancestor (LCA) subtree [21]. The experimental results prove that this strategy can improve the robustness of our model. The model achieves the best balance between noisy words and relevant words when *N* is set to 2.

A single GCN model usually depends highly on correct parse trees to extract crucial information from sentences, while existing parsing algorithms produce imperfect trees in many cases. To further improve the robustness of our mode, we apply a Bi-LSTM network to obtain contextual information about word order or disambiguation. The compound model can better leverage local word patterns regardless of parsing quality.

In summary, we propose a GCN-based model in this paper to extract CPIs. We evaluated our model on the ChemProt corpus, which is the benchmark data set for CPI extraction. To the best of our knowledge, this is the first study to use a GCN encoding syntactic graph for CPI extraction.

## Methods

### Overview

**Figure 1:**
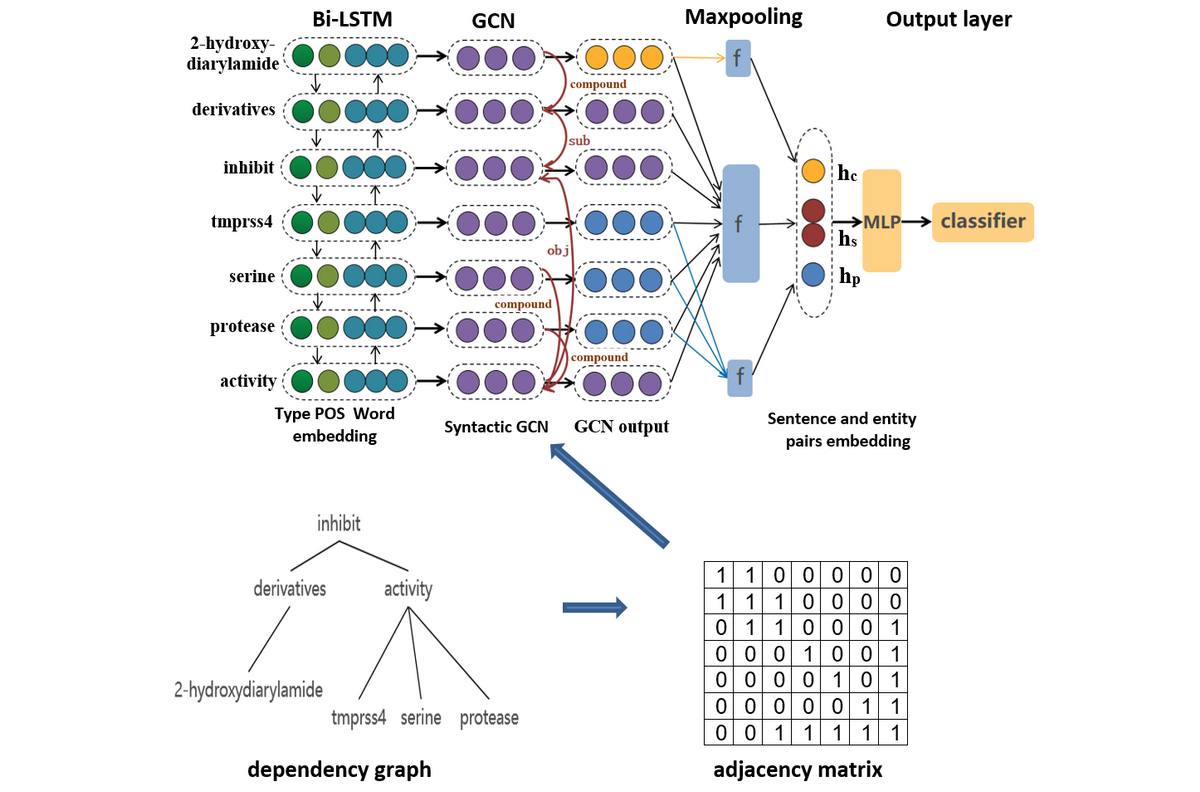
The overall architecture of our model. Bi-LSTM: bi-directional long short-term memory; GCN: graph convolutional network; POS: part-of-speech; MLP: multilayer perceptron; sub: subject; obj: object; hc: representation of chemical; hs: representation of sentence; hp: representation of protein; f: max-pooling function.

The overall architecture of our model is presented in Figure 1. Our model contains three parts: the Bi-LSTM layer, the GCN layer, and the classification layer. In the model, a Bi-LSTM layer is applied first to capture local word patterns and output the representation of each word within the whole sentence. Subsequently, the contextualized representation and the dependency graph (with two directly attached dependencies) of input sentences are fed into the GCN layer to integrate dependency information into word representations. After that, a max-pooling layer is applied to generate the representation of the sentence and two target entities from word representations. Finally, these representations are concatenated and fed into a multilayer perceptron (MLP) for softmax classification. In the following section, we will introduce our model in detail.

### The Bi-LSTM Layer

We adopt a Bi-LSTM layer to capture contextual information about word order and reduce the impact of parsing errors in our model. The Bi-LSTM layer is applied on the whole sentence to learn the representation of each word. Bi-LSTM can capture more comprehensive features by dealing with the input sequence from forward and backward directions, compared with unidirectional LSTM; it is the combination of the forward LSTM and backward LSTM.

In the ChemProt corpus, some entities contain multiple types of words, especially the relation type “PART_OF,” which means one entity is part of another type of entity within a relation entity pair. For example, “thiazide-sensitive sodium-chloride cotransporter” is a gene entity, and “sodium-chloride” is a chemical entity. To reduce this interference, we apply prior knowledge of the entity type as a feature to improve CPI extraction.

The input of the Bi-LSTM layer consists of three parts, including word embedding, part-of-speech (POS) embedding and entity type embedding. Given a sentence *S = {w_1_,w_2_,…,w_n_}*, the POS sequence *P = {p_1_,p_2_,…,p_n_}* can be obtained by the Stanford CoreNLP toolkit [22], where *w_i_* is the i-th word in a sentence and *p_i_* is its POS. We obtain the sequence of entity types *T = {t_1_,t_2_,…,t_n_}* through the index information of the entity pairs in a sentence. We tagged entity tokens “chemical” or “gen” and other words “O.” The word embedding is initialized with pretrained word embedding, which is obtained by FastText [23]. POS and entity type embedding are initialized randomly. The input of the model is denoted as follows:

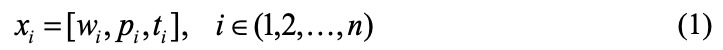



For each token *x_i_*, the forward LSTM and backward LSTM consider the contextual information before and after it, respectively. The final output is the concatenation of the two directions. The Bi-LSTM calculation process is presented as follows:

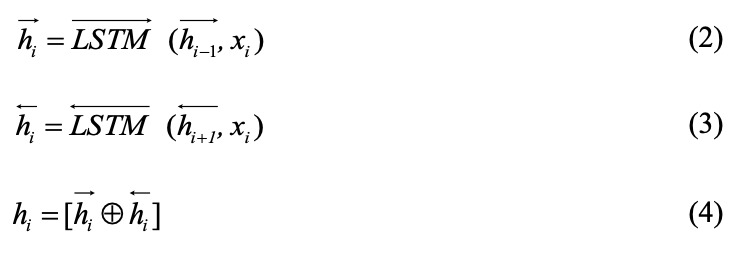



where 
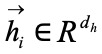
 and 
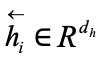
 denote the hidden states of the forward and backward LSTM of *x_i_*, respectively. 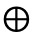 denotes concatenation operation.

### The GCN Layer

GCNs can learn a state embedding, which contains the information of a neighborhood for each node in a graph. It has been proven that models or dependency-based models are very effective in relation extraction by capturing long-range syntactic relations [24-26]. In our model, we apply a GCN to improve the performance of CPI extraction by utilizing the dependency parse trees of the input sentences. In order to reduce the influence of noisy words in long sentences, we further apply a pruning strategy on the dependency trees to remove irrelevant words while maximally keeping crucial content.

Given a sentence, we first apply the Stanford CoreNLP toolkit to get its dependency tree, which is considered as an undirected graph. Then, we apply a path-centric pruning strategy and retain two directly attached words around the shortest path at the LCA of the two entities [20]. After that, we convert the subgraph into an adjacency matrix A. If there is a dependency relation between node *i* and *j*, is assigned with a value of 1. Finally, we apply a GCN over the output of Bi-LSTM and adjacency matrix A to get an updated hidden representation of *h_i_*. This can be represented as shown in formula 5. In an L-layer GCN, if we use 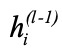 as the input vector and 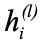 as the output vector for node *i* at the l-th layer, the graph convolution operation of the l-th layer can be represented as shown in formula 6.

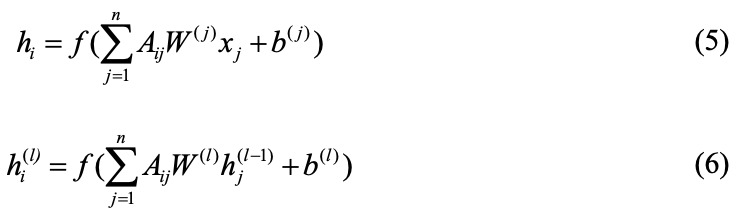



where *W^(j)^* and *W^(l)^* are weight linear transformations, *b^(j)^* and *b^(l)^* are bias terms, and *f* is a nonlinear function (eg, a rectified linear unit [ReLU]). We could obtain the hidden representation of each token directly influenced by its neighbors no more than *L* edges apart in the dependency trees after applying an L-layer GCN over word vectors. To avoid a sentence representation favoring high-degree nodes regardless of the information carried in the node and to transfer information in 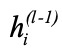 to 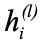, we normalized the activations in the graph convolution before feeding it through a nonlinearity, and added self-loops to each node in the graph:

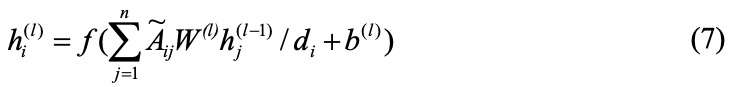



where 
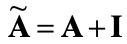
 . *I* is the *n* × *n* identity matrix, and 
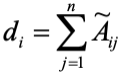
 is the degree of token *i* in the resulting graph.

### The Output and Classification Layer

The CPI extraction can be regarded as a classification problem. Given a sentence *S = {w_1_,w_2,_...,w_n_}* where *w_i_* is the *i*-th token, let *S_c_ = {w_c1_,w_c2_,...,w_cn_}* and *S_p_ = {w_p1_,w_p2_,...,w_pn_}* denote chemical sequence and protein sequence, respectively. The goal of CPI extraction is to predict the relation rR hold between the chemical *S_c_* and gen *S_p_*; otherwise, “no relation” is declared. After the Bi-LSTM and GCN layers, we can obtain the hidden representation of each token, which is influenced by not only local word patterns but also long-range words. To utilize these word representations for relation extraction, we mapped from *h^(L)^* (*n* output vectors) to the sentence vector *h_sent_*. The information close to entity tokens in the dependency trees is generally important in relation classification. Therefore, we also apply a max-pooling function to obtain entity pair representations *h_c_* and *h_p_* from *h^(L)^* as follows:

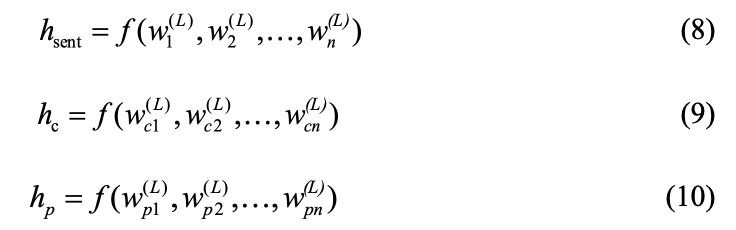



where 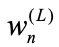 denotes the output after L-layer GCN, and *f* denotes a max-pooling function.

Then, we connect sentence representation with entity representation [27,28] as a new representation, and feed it into a feed-forward neural network (FFNN) inspired by relational reasoning works:

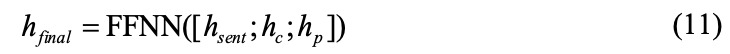



Finally, we apply a linear layer followed by a softmax operation over the final representation *h_final_* to obtain a probability distribution over chemical-protein relations and the computation is shown as follows:

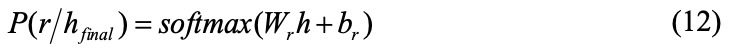



where *W_r_* and *b_r_* are trainable parameters, and *r* is relation type.

### Evaluation Metrics

In experiments, the Micro-average F-score is applied to evaluate the performance of our model, which is a harmonic mean of *P* and *R*, where *P* denotes precision and *R* denotes recall:

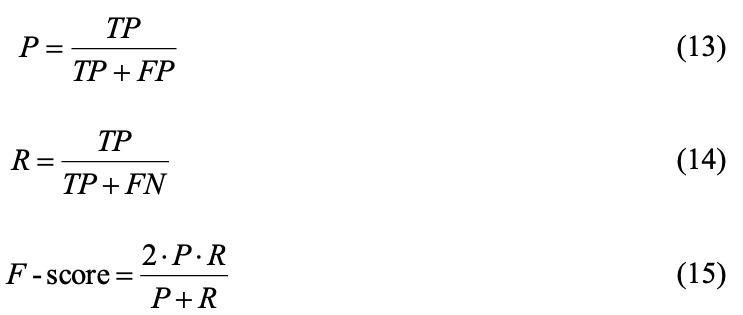



*TP*, *FN*, and *FP* denote true positive, false negative, and false positive, respectively.

## Results

### Data Retrieval and Preprocessing

**Figure 2:**
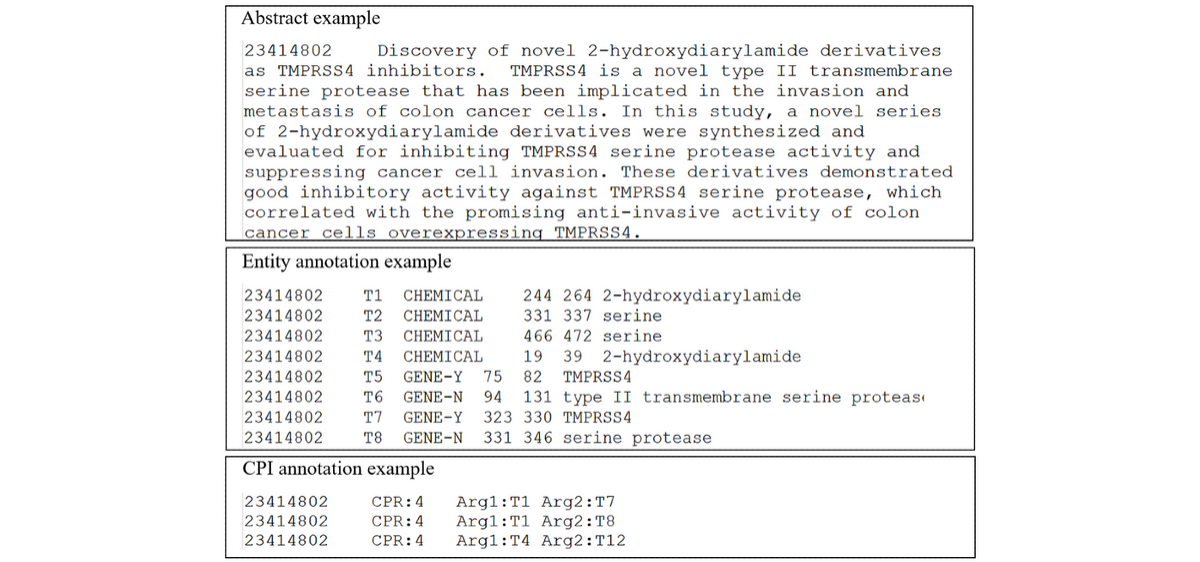
Examples of the ChemProt corpus. CPI: chemical-protein interaction.

CPI extraction aims to classify whether a semantic relation that holds between the chemical and protein entity pairs within a sentence or document. The BioCreative VI ChemProt task delivered the corpus as a manually annotated CPI data set that consists of training, development, and test sets. Each set includes the abstracts, entities, and relations files. Figure 2 provides an example of the three files from the ChemProt training set.

The abstracts file provides the article identifier, title, and abstract document for each article. The entities file consists of the PubMed Unique Identifier (PMID), entity number, type of entity mentions, start and end character offset, and text string of entity mention. The relations file is composed of the PMID, CPI relation class, evaluation type, and CPI relation and interactor arguments. In the ChemProt corpus, there are 10-type relation classes, and each relation class includes one or multiple relation types (Table 1). Although there are 10-type relation classes in ChemProt corpus, only five are used for evaluation purposes (ie, CPR:3, CPR:4, CPR:5, CPR:6, and CPR:9). Table 2 shows the statistics of the ChemProt corpus.

The original corpus consists of PubMed abstracts from biomedical literature in which more than 98% of relation entity pairs within a sentence [8]. Therefore, we neglected the cross-sentence entity pairs and conducted experiment at the sentence level. For CPI extraction, we took some preprocessing steps on the original corpus. First, we split abstracts into sentences and only retained the sentences that contained the relational entity pairs. Then, we reassigned the training set and developing set with a ratio of 9:1. Finally, we replaced each digit string that was not an entity substring with a particular “num” tag.

**Figure 3:**
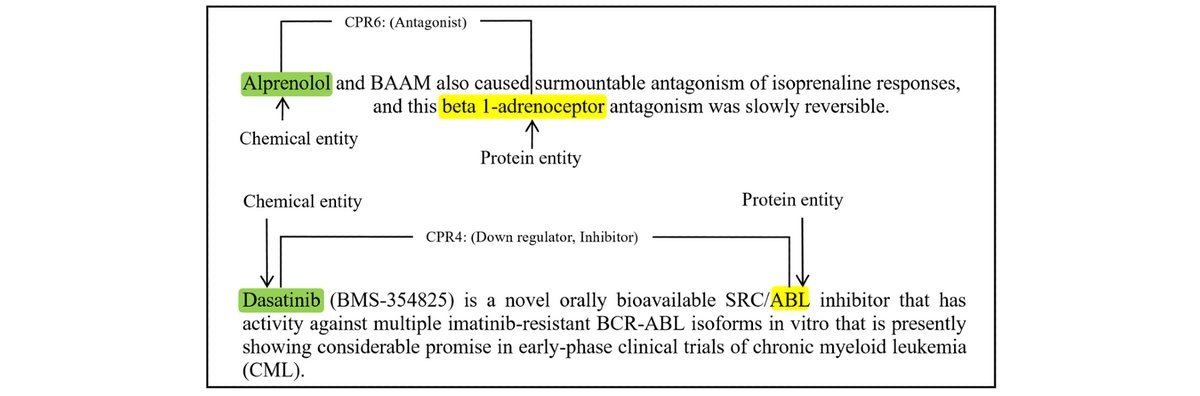
Illustrative examples of chemical-protein relation (CPR) classes.

Figure 3 gives two illustrative examples of CPI extraction. In the first example, the sentence “Alprenolol and BAAM also caused surmountable antagonism of isoprenaline responses, and this beta 1-adrenoceptor antagonism was slowly reversible.” contains a relational entity pair. To accurately extract the CPI, we need to first detect the chemical entity “Alprenolol” and protein entity “beta 1-adrenoceptor,” and then classify the interaction as the CPR:6 class. The second example is a long and complex sentence. It is more difficult for the relation classifier to extract the interaction between the chemical and protein entities. Our model aims to predict the interactions, and the output is the relation type of chemical-protein entity pairs as shown in Figure 3.

**Table 1:** The chemical-protein relation (CPR) groups.

Group	Evaluated in the BioCreative VI ChemProt shared task?	ChemProt relations
CPR:1	No	PART_OF
CPR:2	No	REGULATOR|DIRECT_REGULATOR|INDIRECT_REGULATOR
CPR:3	Yes	UPREGULATOR|ACTIVATOR|INDIRECT_UPREGULATOR
CPR:4	Yes	DOWNREGULATOR|INHIBITOR|INDIRECT_DOWNREGULATOR
CPR:5	Yes	AGONIST|AGONIST-ACTIVATOR|AGONIST-INHIBITOR
CPR:6	Yes	ANTAGONIST
CPR:7	No	MODULATOR|MODULATOR-ACTIVATOR|MODULATOR-INHIBITOR
CPR:8	No	COFACTOR
CPR:9	Yes	SUBSTRATE|PRODUCT_OF|SUBSTRATE_PRODUCT_OF
CPR:10	No	NOT

**Table 2:** Statistics of the ChemProt corpus.

Annotations	Data set		
	Training, n	Development, n	Test, n
Document	1020	612	800
Chemicals	13,017	8004	10,810
Proteins	12,752	7567	10,019
CPR^a^:3	768	550	665
CPR:4	2254	1094	1661
CPR:5	173	116	195
CPR:6	235	199	293
CPR:9	727	457	644
Evaluated CPIs^b^	4157	2416	3458
Evaluated CPIs in one sentence	4122	2412	3444

^a^CPR: chemical-protein relation.

^b^CPI: chemical-protein interaction.

### Experimental Settings

In this work, FastText [23] was used to pretrain word embedding on the ChemProt corpus. Before the experiments, we set the range of parameters based on experience, then tuned the parameters on the development set by using grid search to determine the optimal parameters, and finally selected the best model of parameters that were optimal for evaluation on the test set. Without overfitting, the best model generally can achieve the best performance (the highest F-score) on the development set. The detailed tune range and hyperparameter values are listed in Table 3.

### Comparison of Different Pruning Distances

To obtain the best pruning distance, we experimented with *N*{0,1,2,3,∞} on the ChemProt corpus—*N*=0 corresponds to pruning the tree down to the path; *N*=1 keeps all nodes that are directly attached to the path; *N*=2,3 means holding words up to distance 2 and 3 away from the dependency path in the LCA subtree; and *N*=∞ retains the entire LCA subtree.

**Figure 4:**
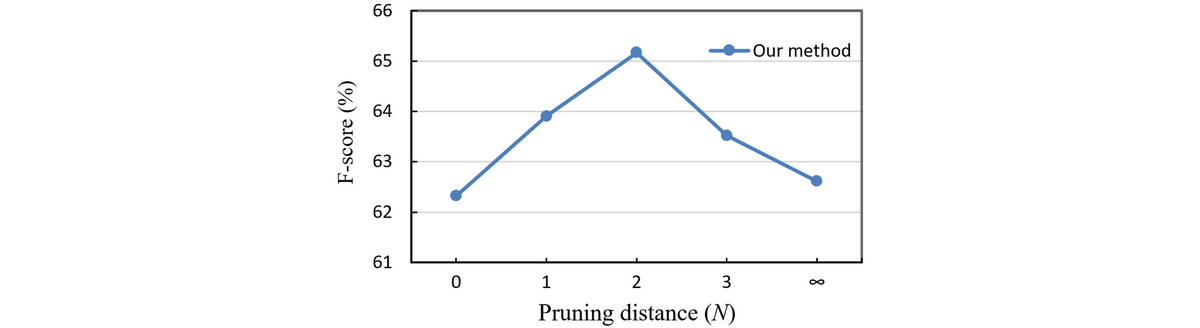
Comparison of different pruning distances.

As shown in Figure 4, the performance of our model reaches its peak and outperforms other pruning distance at *N*=2. This confirms that pruning too aggressively (*N*=0,1) could lead to a loss of crucial information while retaining too many irrelevant words (*N*=3) also decreases model performance due to the interference of irrelevant information. When *N*=2, the model achieves the best balance between including relevant and irrelevant information.

**Table 3:** Hyperparameter setting.

Hyperparameter	Tuned range	Optimal
Word embedding dimension	[100,200,300]	200
POS^a^ embedding dimension	[10,20,30,40]	20
Entity type embedding dimension	[40,50,60,70,80]	60
GCN^b^ hidden units	[100,200,300]	200
LSTM^c^ hidden units	[100,200,300]	200
Learning rate	[0.1,0.2,0.3,0.4]	0.3
Dropout rate	[0.4,0.5,0.6]	0.5

^a^POS: part-of-speech.

^b^GCN: graph convolutional network.

^c^LSTM: long short-term memory.

### Comparison of Different Embedding Features

Table 4 shows the effectiveness of different embedding features, including word embedding, entity type embedding, and POS embedding. The model achieves an F-score of 59.56% when only using word embedding. When POS and word embedding are combined, the F-score increases to 60.69%. When the entity type and word embedding are combined, the F-score increases to 62.52% (an increase of 2.96%). Furthermore, when both entity type and POS embedding are integrated with word embedding, the F-score improves to 65.17%. The results suggest that the main contributor to performance is prior knowledge of the entity type. This confirms the validity of the entity type in CPI extraction. The POS embedding is also valuable to the model.

**Table 4:** Performance evaluation of different embedding features.

Embedding feature	Precision (%)	Recall (%)	F-score (%)	Δ (%)
Word	57.64	61.62	59.56	—^a^
Word+POS^b^	58.49	63.06	60.69	+1.13
Word+Entity type	64.06	61.05	62.52	+2.96
Word+POS+Entity type	63.79	66.62	65.17	+5.61

^a^Not applicable.

^b^POS: part-of-speech.

### Comparison With the Baseline Method

Different single models and their ensemble models are compared with each other in this section. As shown in Table 5, all ensemble models perform better than all single models, and the GCN+Bi-LSTM model performs better than the Bi-LSTM+CNN model. The results indicate that ensemble models can generally capture more information than single models. In terms of overall performance, the precision, recall, and F-score of the Bi-LSTM+GCN model are higher than those of the Bi-LSTM+CNN model. Our model can fully capture the overall information of the sentence by combining sequence structure information and syntactic information, while the Bi-LSTM+ CNN model could only obtain sequence structure information, which confirms the effectiveness of the GCN model in CPI extraction.

**Table 5:** Comparison with the baseline method.

Model			Precision (%)		Recall (%)		F-score (%)
**Single models**							
	CNN^a^	42.47		69.43		52.70	
	GCN^b^	48.77		63.69		55.24	
	Bi-LSTM^c^	60.59		60.34		60.46	
**Ensemble models**							
	Bi-LSTM+CNN	57.77		64.73		61.05	
	Bi-LSTM+GCN (our model)	63.79		66.62		65.17	

^a^CNN: convolutional neural network.

^b^GCN: graph convolutional network.

^c^Bi-LSTM: bi-directional long short-term memory.

## Discussion

The experimental results suggest that our model can effectively extract CPIs; it is better at learning semantic and syntactic information from sentences compared to other models. Additionally, the pruning strategy can alleviate the influence of irrelevant words in long sentences in biomedical literature, by only retaining *N* away tokens from the dependency path in the LCA subtree.

### Comparison With Prior Work

A comparison of our model with other existing methods on the ChemProt corpus is shown in Table 6. It can be found that neural network–based methods perform better than traditional machine learning–based methods, and our method achieves the highest F-score of 65.17%.

**Table 6:** Comparison with other existing methods.

Model	Precision (%)	Recall (%)	F-score (%)
Verga et al [12]	48.00	54.10	50.80
Matos [29]	57.38	47.22	51.81
Liu et al [11]	57.4	48.7	52.7
Lung et al [30]	63.52	51.21	56.71
Corbett and Boyle [13]	62.97	62.20	62.58
Mehryary et al [9]	59.05	67.76	63.10
Peng et al [14]	72.66	57.35	64.10
Our model	63.79	66.62	65.17

Lung et al [30] used machine learning methods to integrate the semantic and dependency graph features through a three-stage model. They achieved an F-score of 56.71%. Similarly, Corbett and Boyle [13] used pretrained LSTM and Bi-LSTM to extract CPIs in two stages and achieved a higher F-score of 61.5%. A particular feature of their system was the usage of unlabeled data both to pretrain word embedding and pretrain LSTM layers in the neural network.

Verga et al [12] applied attention mechanisms in their model. They synthesized convolutions and self-attention to extract CPIs. Liu et al [11] achieved an F-score of 52.7% by synthesizing GRU and attention pooling. The results of word-level attention weights in the model of Liu et al [11] showed that attention mechanism is effective in selecting the most important trigger words when trained with semantic relation labels without the need of semantic parsing and feature engineering.

Mehryary et al [9] employed an ensemble system that combined the results of SVM and LSTM, and they achieved a competitive result. Peng et al [14] utilized more external features. They stacked SVM, CNN, and RNN models, and combined the outputs of the three systems by either majority voting or stacking. They achieved the best F-score of 64.10% in the BioCreative VI ChemProt shared task. Our model synthesized Bi-LSTM and GCN and achieved an improvement of 1.07% in F-score over the system of Peng et al [14]. We further performed significance tests with *P*<.05 indicating significance. The *P* value of Peng et al [14] and our model is less than .001. It indicates that the improvement of 1.07% in F-score is significant.

### Results Analysis

The experimental results indicate that the GCN module is valuable in CPI extraction. It can extract CPIs from biomedical texts with syntactic graph representations. It might be also efficient in other biomedical tasks by utilizing the sentence parse structure. By comparing different pruning distance, we revealed that the length of sentence also plays an important role in relation extraction. The noisy words that are irrelevant to relations might hamper the performance of the extractor.

GCNs can learn effective representation for relation extraction. However, a single GCN model could not capture the contextual information of word order. Additionally, GCN highly depends on correct parse trees to extract information from sentences, while existing parsing algorithms produce imperfect trees in many cases. To resolve these issues and improve the robustness of our model, we applied Bi-LSTM to generate contextualized representation and feed it into the GCN layer. The results confirm that the ensemble model of GCN and Bi-LSTM is validated for CPI extraction.

### Contributions

The model we proposed in this paper aims to extract CPI and achieve state-of-the-art performance on the ChemProt corpus. Our main contributions are as follows.

We proposed a novel neural model based on a GCN for CPI extraction, which can capture long-range syntactic information by utilizing the dependency structure of the input sentence. To improve the robustness, we applied a path-centric pruning strategy to remove irrelevant words without damaging crucial content on the dependency trees. Through the pruning strategy, the influence of noisy words can be reduced, thereby further improving the performance of the model. Furthermore, a Bi-LSTM layer is utilized to better leverage local word patterns regardless of parsing quality.

Our model can automatically extract CPIs from a large amount of biomedical literature, which can save significant labor force and resources. Abundant biological entity relations can deliver useful chemicals for some diseases and save time by optimizing the drug development cycle, thereby helping pharmacists discover drugs. Furthermore, the knowledge graph generally contains rich, structured knowledge and has been widely used in natural language processing applications, such as search engines and question answering systems. However, the rapidly increasing volume of information requires refinement in the coverage of knowledge graphs. CPI extraction can help researchers to efficiently acquire biomedical knowledge, which can enrich the information needed for knowledge graph construction.

### Conclusions

We proposed a novel model based on a GCN to extract CPI. The GCN module can encode syntactic information over the dependency graphs of input sentences. To reduce the impact of noisy words, our model only retains tokens that are up to a distance of N=2 away from the dependency path in the LCA subtree. Additionally, it applies Bi-LSTM to generate a contextualized representation and feed it into the GCN layer to resolve parsing errors and improve the robustness of the model. The experimental results demonstrated that our model achieves state-of-the-art performance. We plan to further improve our model and apply our method to extract other biomedical relation entity pairs.

## Abbreviations

Bi-LSTM: bi-directional long short-term memory
CNN: convolutional neural network
CPI: chemical-protein interaction
CPR: chemical-protein relation
FFNN: feed-forward neural network
FN: false negative
FP: false positive
GCN: graph convolutional network
GRU: gated recurrent units
LCA: lowest common ancestor
LSTM: long short-term memory
MLP: multilayer perceptron
PMID: PubMed Unique Identifier
POS: part-of-speech
ReLU: rectified linear unit
SVM: support vector machine
TP: true positive
